# Lattices in Tate modules

**DOI:** 10.1073/pnas.2113201118

**Published:** 2021-11-30

**Authors:** Bjorn Poonen, Sergey Rybakov

**Affiliations:** ^a^Department of Mathematics, Massachusetts Institute of Technology, Cambridge, MA 02139-4307;; ^b^Laboratory 13, Institute for Information Transmission Problems of the Russian Academy of Sciences, Moscow 127051, Russia;; ^c^Interdisciplinary Scientific Center J.-V. Poncelet, Moscow 119002, Russia

**Keywords:** abelian variety, Tate module, endomorphism, Dieudonné module

## Abstract

Refining a theorem of Zarhin, we prove that, given a *g*-dimensional abelian variety *X* and an endomorphism *u* of *X*, there exists a matrix $A\in {\text{M}}_{2g}(\mathbb{Z})$ such that each Tate module $T_{\ell }X$ has a ${\mathbb{Z}}_{\ell }$-basis on which the action of *u* is given by *A*, and similarly for the covariant Dieudonné module if over a perfect field of characteristic *p*.

## Introduction

Let *X* be an abelian variety of dimension *g* over a field *k* of characteristic p≥0. Let End X be its endomorphism ring. Let ${\text{End}}^{{}^{\circ}}X≔(\text{End}\;X)⊗\mathbb{Q}$. Define Tate modules

$$\begin{array}{l} T_{\ell }=T_{\ell }X≔\underset{n}{\underset{\leftarrow }{\lim\;}}X[\ell^{n}](\bar{k})\;\;\text{for}\;\text{each}\;\text{prime}\;\ell \neq p\\ V_{\ell }=V_{\ell }X≔T_{\ell }X{⊗}_{{\mathbb{Z}}_{\ell }}{\mathbb{Q}}_{\ell }\;\;\text{for}\;\text{each}\;\text{prime}\;\ell \neq p\\ T=TX≔\prod_{\ell \neq p}T_{\ell }X\\ V=VX\;≔TX{⊗}_{\mathbb{Z}}\mathbb{Q}≃\prod_{\ell \neq p^{\prime }}\left(V_{\ell }X,T_{\ell }X\right); \end{array}$$

these are free rank 2*g* modules over ${\mathbb{Z}}_{\ell },\;{\mathbb{Q}}_{\ell },\;{\hat{\mathbb{Z}}}^{\left(p\right)}≔\prod_{\ell \neq p}{\mathbb{Z}}_{\ell }$, and

$$A^{\left(p\right)}≔{\hat{\mathbb{Z}}}^{\left(p\right)}{⊗}_{\mathbb{Z}}\mathbb{Q}≃\prod_{\ell \neq p^{\prime }}\left({\mathbb{Q}}_{\ell },{\mathbb{Z}}_{\ell }\right),$$

respectively. If *p* > 0 and *k* is perfect, we have also a *p*-adic analog of $T_{\ell }X$, namely, the *covariant* Dieudonné module ${\text{M}}_{*}(X)$, and we define

$$\begin{array}{l} M≔{\text{M}}_{*}(X)\;\;\;\;\;\;\;\;\;\;\;\;M_{\mathbb{Q}}≔M{⊗}_{\mathbb{Z}}\mathbb{Q}=M[1/p]\\ T_{W}≔T\times M\;\;\;\;\;\;\;\;\;\;\;\;\;\;V_{W}≔T_{W}{⊗}_{\mathbb{Z}}\mathbb{Q}=V\times M_{\mathbb{Q}}; \end{array}$$

these are free rank 2*g* modules over the ring of Witt vectors W≔W(k), its fraction field

$$K≔W{⊗}_{\mathbb{Z}}\mathbb{Q}=W[1/p],$$

the product ${\hat{\mathbb{Z}}}^{\left(p\right)}\times W$, and

$$A_{W}≔({\hat{\mathbb{Z}}}^{\left(p\right)}\times W){⊗}_{\mathbb{Z}}\mathbb{Q}=A\times \mathbb{Q},$$

respectively.

Definition 1.1: Given rings R⊆R′ and corresponding modules L⊆L′, say that *L* is an *R*-lattice in L′ if *L* has an *R*-basis that is an R′-basis for L′.

Zarhin (ref. [1], Theorem 1.1) proved that, given $u\in {\text{End}}^{{}^{\circ}}X$, there exists a matrix $A\in {\text{M}}_{2g}(\mathbb{Q})$ such that, for every ℓ≠p, there is a ${\mathbb{Q}}_{\ell }$-basis of $V_{\ell }$ on which the action of *u* is given by *A*; equivalently, there exists a *u*-stable ℚ-lattice in the $\left(\prod_{\ell \neq p}{\mathbb{Q}}_{\ell }\right)$-module $\prod_{\ell \neq p}V_{\ell }$. Our main theorem refines this, as follows.

Theorem 1.2. *Let* $u\in {\text{End}}^{{}^{\circ}}X$.

(a) *There exists a u-stable* ℚ-*lattice* V⊂V.
(b) *There exists a u-stable* ℤ-*lattice* T⊂T.
(c) *If p* > 0 *and k is perfect, then there exists a u-stable* ℚ-*lattice* $V\subset V_{W}$.
(d) *If p* > 0 *and k is perfect, then there exists a u-stable* ℤ-*lattice* $T\subset T_{W}$.

The following restatement of (b) answers a question implicit in ref. [1], Remark 1.2.

Corollary 1.3. *Let* u∈End X. *Then there exists a matrix* $A\in {\text{M}}_{2g}(\mathbb{Z})$ *such that, for every* ℓ≠p, *there is a* ${\mathbb{Z}}_{\ell }$*-basis of* $T_{\ell }X$ *on which the action of u is given by A, and such that, if p* > 0 *and k is perfect, there is a W-basis of* ${\text{M}}_{*}(X)$ *on which the action of u is given by A.*

The characteristic 0 case of *Theorem 1.2* can be proved by reducing to the case k=ℂ and taking rational or integral homology (ref. [1], Remark 1.2). But pairs (*X*, *u*) in characteristic *p* > 0 cannot always be lifted to characteristic 0 (ref. [2], Example 14.5), so the general case does not seem to follow easily from this.

## Proof

Lemma 2.1. *Suppose that p* > 0 *and k is perfect. Let L be a finite extension of* ${\mathbb{Q}}_{p}$. *Let N be an* $\left(L{⊗}_{{\mathbb{Q}}_{p}}K\right)$-*module with an automorphism F that is L-linear and K-semilinear with respect to the Frobenius automorphism* ϕ *of K. Then N is free over* $L{⊗}_{{\mathbb{Q}}_{p}}K$.

Proof: The residue field ℓ of *L* is finite, so it has a largest subextension ℓ′ embeddable in *k*. Let L′⊂L be the corresponding unramified extension of ${\mathbb{Q}}_{p}$. Let $I={\text{Hom}}_{{\mathbb{Q}}_{p}-\text{algebras}}(L\prime ,K)$. Then

$$L\prime {⊗}_{{\mathbb{Q}}_{p}}K≃\prod_{i\in I}K.$$

Applying $L{⊗}_{L\prime }$ yields

$$L{⊗}_{{\mathbb{Q}}_{p}}K≃\prod_{i\in I}L_{i},$$

where each *L_i_* is a field since *K* is absolutely unramified and any tensor product $\ell {⊗}_{\ell \prime }k$ is a field. Now $N≃{⊕}_{i\in I}N_{i}$, where each *N_i_* is a *L_i_*-vector space.

The action of ϕ on *K* induces a permutation *π* of *I* that is transitive since $L\prime /{\mathbb{Q}}_{p}$ is Galois with group generated by the Frobenius automorphism. If i∈I and j=π(i), then the compatible actions of ϕ of *K* and *F* on *N* induce compatible isomorphisms $L_{i}\tilde{\to }L_{j}$ and $N_{i}\tilde{\to }N_{j}$ for each *i*, so ${\dim\;}_{L_{i}}N_{i}={\dim\;}_{L_{j}}N_{j}$. It follows, by transitivity of *π*, that ${\dim\;}_{L_{i}}N_{i}$ is independent of *i*. Thus the module $N={⊕}_{i\in I}N_{i}$ is free over $L{⊗}_{{\mathbb{Q}}_{p}}K≃\prod_{i\in I}L_{i}$.

Lemma 2.2. *Let E be a number field contained in* ${\text{End}}^{{}^{\circ}}X$. *Let* O=E∩End X. *Let* h=2(dim X)/[E:ℚ]. *Then*

(i) *The* $\left(E{⊗}_{\mathbb{Q}}{\mathbb{Q}}_{\ell }\right)$-*module* $V_{\ell }$ *is free of rank h.*
(ii) *If p* > 0 *and k is perfect, then the* $\left(E{⊗}_{\mathbb{Q}}K\right)$-*module* $M_{\mathbb{Q}}$ *is free of rank h.*
(iii) *For each* ℓ∤p disc O, *the* $\left(O{⊗}_{\mathbb{Z}}{\mathbb{Z}}_{\ell }\right)$-*module* $T_{\ell }$ *is free of rank h.*
(iv) *The* $\left(E{⊗}_{\mathbb{Q}}A^{\left(p\right)}\right)$-*module* V *is free of rank h.*
(v) *If p* > 0 *and k is perfect, then the* $\left(E{⊗}_{\mathbb{Q}}A_{W}\right)$-*module* $V_{W}$ *is free of rank h.*

Proof:

(i) This is ref. [3], Theorem 2.1.1.
(ii) The following proof is essentially a combination of the proofs of ref. [4], Proposition 1.4.3.9(1) and ref. [3], Theorem 2.1.1. Write $E{⊗}_{\mathbb{Q}}{\mathbb{Q}}_{p}≃\prod_{j}E_{j}$ for some finite extensions *E_j_* of ${\mathbb{Q}}_{p}$. Correspondingly, $M_{\mathbb{Q}}≃{⊕}_{j}M_{j}$. The Frobenius automorphism of the Dieudonné module *M* induces an *E_j_*-linear and *K*-semilinear automorphism of *M_j_*. By *Lemma 2.1*, the $\left(E_{j}{⊗}_{{\mathbb{Q}}_{p}}K\right)$-module *M_j_* is free.
  On the other hand, by ref. [5] (p. 96), Corollary, for any x∈E, the characteristic polynomials of the actions of *x* on $M_{\mathbb{Q}}$ and $V_{\ell }$ are the same. Now repeat the proof of ref. [3], Theorem 2.1.1.
(iii) Fix ℓ∤p disc O, where disc O is the discriminant of O. For each prime *λ* of O dividing ℓ, let $O_{\lambda }\subset E_{\lambda }$ be the completions of O⊂E at *λ*. Since ℓ∤disc O, the ring $O_{\lambda }$ is a discrete valuation ring with fraction field $E_{\lambda }$, and
  $$E{⊗}_{\mathbb{Q}}{\mathbb{Q}}_{\ell }≃\prod_{\lambda |\ell }E_{\lambda }\;\text{and}\;O{⊗}_{\mathbb{Z}}{\mathbb{Z}}_{\ell }≃\prod_{\lambda |\ell }O_{\lambda }.$$
  These induce decompositions
  $$V_{\ell }=\prod_{\lambda |\ell }V_{\lambda }\;\text{and}\;T_{\ell }=\prod_{\lambda |\ell }T_{\lambda }.$$
  By (i), ${\dim\;}_{E_{\lambda }}V_{\lambda }=h$. Since $T_{\lambda }$ is a torsion-free finitely generated $O_{\lambda }$-module that spans $V_{\lambda }$, it is free of rank *h* over $O_{\lambda }$. Thus $T_{\ell }=\prod_{\lambda |\ell }T_{\lambda }$ is free of rank *h* over $O{⊗}_{\mathbb{Z}}{\mathbb{Z}}_{\ell }≃\prod_{\lambda |\ell }O_{\lambda }$.
(iv) We have $E{⊗}_{\mathbb{Q}}A^{\left(p\right)}=\prod^{\prime }(E{⊗}_{\mathbb{Q}}{\mathbb{Q}}_{\ell },O{⊗}_{\mathbb{Z}}{\mathbb{Z}}_{\ell })$, so (iv) follows from (i) and (iii).
(v) Similarly, this follows from (i), (ii), and (iii). __

Proof of Theorem 1.2:

(a) We work in the category of abelian varieties over *k* up to isogeny. By ref. [1], Theorem 2.4, *u* is contained in a subring of ${\text{End}}^{{}^{\circ}}X$ isomorphic to $\prod_{i}{\text{M}}_{r_{i}}(E_{i})$ for some number fields *E_i_*. Then *X* is isogenous to $\prod Y_{i}^{r_{i}}$ for some abelian varieties *Y_i_* with $E_{i}\subseteq {\text{End}}^{{}^{\circ}}Y_{i}$. If we can find an *E_i_*-stable ℚ-lattice $V_{i}\subset VY_{i}$ for each *i*, then we may take $V=\prod V_{i}^{r_{i}}$. In other words, we have reduced to the case that $u\in E\subseteq {\text{End}}^{{}^{\circ}}X$ for some number field *E*. By *Lemma 2.2* (iv),
  $$V=P{⊗}_{\mathbb{Q}}(E{⊗}_{\mathbb{Q}}A^{\left(p\right)})$$
  for some ℚ-vector space *P*. Then $V≔P{⊗}_{\mathbb{Q}}E$ is a *u*-stable ℚ-lattice in V.
(b) Given u∈End X, choose *V* as in (a). We have
  $$\mathbb{Q}\cap {\hat{\mathbb{Z}}}^{\left(p\right)}=\mathbb{Z}[1/p],$$
  which we interpret as ℤ if *p* = 0. Then V∩T is a ℤ[1/p]-lattice in T. Since ℤ[u]⊂End X is a finite ℤ module, the ℤ[u]-submodule generated by any ℤ[1/p]-basis of V∩T is a *u*-stable ℤ-lattice.
(c) As in the proof of (a), we reduce to the case in which $u\in E\subseteq {\text{End}}^{{}^{\circ}}X$ for some number field *E*. By *Lemma 2.2* (v),
  $$V_{W}=P{⊗}_{\mathbb{Q}}(E{⊗}_{\mathbb{Q}}A_{W})$$
  for some ℚ-vector space *P*. Then $V≔P{⊗}_{\mathbb{Q}}E$ is a *u*-stable ℚ-lattice in $V_{W}$.
(d) Let *V* be as in (c). We have $\mathbb{Q}\cap ({\hat{\mathbb{Z}}}^{\left(p\right)}\times W)=\mathbb{Z}$. Then $V\cap T_{W}$ is a *u*-invariant ℤ-lattice in $T_{W}$.

## Generalizations and Counterexamples

In *Theorem 1.2*, suppose that, instead of fixing one endomorphism *u*, we consider a ℚ-subalgebra $R\subset {\text{End}}^{{}^{\circ}}X$ (or subring R⊂End X) and ask for an *R*-stable ℚ-lattice (respectively, ℤ-lattice), that is, one that is *r* stable for every r∈R.

(1) If *R* is contained in a subring of ${\text{End}}^{{}^{\circ}}X$ isomorphic to $\prod_{i}{\text{M}}_{r_{i}}(E_{i})$ for some number fields *E_i_*, then the proof of *Theorem 1.2* shows that an *R*-stable lattice exists.
(2) Serre observed that if *X* is an elliptic curve such that ${\text{End}}^{{}^{\circ}}X$ is a quaternion algebra, then for $R={\text{End}}^{{}^{\circ}}X$, there is no *R*-stable ℚ-lattice in any $V_{\ell }$, since *R* cannot act on a two-dimensional ℚ-vector space.
(3) If *R* is assumed to be commutative, then the conclusions of *Theorem 1.2* can still fail. For example, suppose that *Y* is an elliptic curve such that ${\text{End}}^{{}^{\circ}}Y$ is a quaternion algebra *B*, and $X=Y^{2}$, and
  $$\begin{array}{l} R=\left\{\left(\begin{array}{cc} a & b\\ 0 & a \end{array}\right):a\in \mathbb{Q}\;\;\text{and}\;b\in B\right\}\\ \subset {\text{M}}_{2}(B)={\text{End}}^{{}^{\circ}}X. \end{array}$$
  The ideal $\left(\begin{array}{cc} 0 & B\\ 0 & 0 \end{array}\right)$ has square zero, so *R* is commutative. For each nonzero b∈B, we have
  $$\left(\begin{array}{cc} 0 & b\\ 0 & 0 \end{array}\right)X=0\times Y,\;\text{so}\;\left(\begin{array}{cc} 0 & b\\ 0 & 0 \end{array}\right)VX=0\times VY,$$
  which is of rank 2.
  Suppose that there is an *R*-stable ℚ-lattice *V* in VX. Let U≔V∩(0×VY), which is a ℚ-vector space of dimension at most 2. Then, for every nonzero b∈B, the image $\left(\begin{array}{cc} 0 & b\\ 0 & 0 \end{array}\right)V$ is a 2-dimensional ℚ-lattice in 0×VY, contained in *U*, and hence equal to *U*. Thus we obtain a ℚ-linear injection
  $$\left(\begin{array}{cc} 0 & B\\ 0 & 0 \end{array}\right),\to \text{Hom}(V/U,U)\subset \text{End}\;V.$$
  It is an isomorphism since
  $$\dim\;\left(\begin{array}{cc} 0 & B\\ 0 & 0 \end{array}\right)=4=\dim\;\text{Hom}(V/U,U).$$
  Since ${\dim\;}_{\mathbb{Q}}\left(\begin{array}{cc} 0 & b\\ 0 & 0 \end{array}\right)V=2$ for each nonzero b∈B, we have ${\dim\;}_{\mathbb{Q}}f(V)=2$ for each nonzero *f* in Hom(V/U,U)⊂End V, which is absurd. Thus there is no *R*-stable ℚ-lattice in VX.

## Data Availability

There are no data underlying this work.
